# Risk factors for perineal and vaginal tears in primiparous women – the prospective POPRACT-cohort study

**DOI:** 10.1186/s12884-020-03447-0

**Published:** 2020-12-02

**Authors:** Markus Harry Jansson, Karin Franzén, Ayako Hiyoshi, Gunilla Tegerstedt, Hedda Dahlgren, Kerstin Nilsson

**Affiliations:** 1 Department of Obstetrics and Gynecology, Örebro University Hospital, Örebro, Sweden; 2 School of Medical Sciences, Faculty of Health and Medicine, Örebro University, SE-701 82 Örebro, Sweden; 3 Unit of Obstetrics and Gynecology, CLINTEC, Karolinska University Hospital at Huddinge, Karolinska Institutet, Stockholm, Sweden; 4 Department of Surgery, Örebro University Hospital, Örebro, Sweden

**Keywords:** High obstetric vaginal tear, Obstetric anal sphincter injuries, Perineal tears, Risk factors, Second-degree perineal tears

## Abstract

**Background:**

The aim of this study was to estimate the incidence of second-degree perineal tears, obstetric anal sphincter injuries (OASI), and high vaginal tears in primiparous women, and to examine how sociodemographic and pregnancy characteristics, hereditary factors, obstetric management and the delivery process are associated with the incidence of these tears.

**Methods:**

All nulliparous women registering at the maternity health care in Region Örebro County, Sweden, in early pregnancy between 1 October 2014 and 1 October 2017 were invited to participate in a prospective cohort study. Data on maternal and obstetric characteristics were extracted from questionnaires completed in early and late pregnancy, from a study-specific delivery protocol, and from the obstetric record system. These data were analyzed using unadjusted and adjusted multinomial and logistic regression models.

**Results:**

A total of 644 women were included in the study sample. Fetal weight exceeding 4000 g and vacuum extraction were found to be independent risk factors for both second-degree perineal tears (aOR 2.22 (95% CI: 1.17, 4.22) and 2.41 (95% CI: 1.24, 4.68) respectively) and OASI (aOR 6.02 (95% CI: 2.32, 15.6) and 3.91 (95% CI: 1.32, 11.6) respectively). Post-term delivery significantly increased the risk for second-degree perineal tear (aOR 2.44 (95% CI: 1.03, 5.77), whereas, maternal birth positions with reduced sacrum flexibility significantly decreased the risk of second-degree perineal tear (aOR 0.53 (95% CI 0.32, 0.90)). Heredity of pelvic floor dysfunction and/or connective tissue deficiency, induced labor, vacuum extraction and fetal head circumference exceeding 35 cm were independent risk factors for high vaginal tears (aOR 2.32 (95% CI 1.09, 4.97), 3.16 (95% CI 1.31, 7.62), 2.53 (95% CI: 1.07, 5.98) and 3.07 (95% CI 1.5, 6.3) respectively).

**Conclusion:**

The present study corroborates previous findings of vacuum extraction and fetal weight exceeding 4000 g as risk factors of OASI. We found that vacuum extraction is a risk factor for second-degree tear, and vacuum extraction, fetal head circumference exceeding 35 cm and heredity of pelvic floor dysfunction and/or connective tissue deficiency were associated with increased risk of high vaginal tears. These findings have not been documented previously and should be confirmed by additional studies.

## Background

Perineal tears affect about 80% of women during childbirth, with primiparous women being affected more frequently than multiparous women [1, 2]. The rate of second-degree perineal tears, which involves the vagina and/or perineal muscle, has been reported to be 35.1–78.3% among primiparous women and 34.8–39.6% among multiparous women [1–3], while third- and fourth-degree tears, which involve varying degrees of injury to the anal sphincters, occur in 5.1–8.3% of primiparous women and 1.8–2.8% of multiparous women [1, 2, 4, 5]. Between 1990 and 2016, the incidence of third- and fourth-degree perineal tears among primiparous women in Sweden rose from 2.9 to 5.1% [6].

Obstetric anal sphincter injuries (OASI) are the largest obstetric risk factor for developing anal incontinence in women [7], so these tears merit particular attention. However, although less attention has been paid, second-degree tears alone may impair sexual function [8] and increase the risk of future pelvic organ prolapse [9], and high vaginal tears have been associated with increased risk for levator muscle avulsion [10]. But the incidence and risk factors of these tears have been poorly investigated.

Various interventions have been attempted to prevent perineal tears, but few have been proven to reduce the incidence of severe perineal tears. There is moderate-quality evidence that warm compresses applied to the perineum during delivery and perineal massage can reduce the risk of OASI [11]. Episiotomy has been shown to be protective against OASI in instrumental vaginal delivery [12, 13], but in spontaneous vaginal delivery the risk of severe perineal trauma is lower when episiotomy is used restrictively rather than routinely [14]. Randomized controlled studies have not shown any advantage of manual perineal support in reducing OASI [11]. An educational program developed in Finland including a specific technique of manual perineal support and mediolateral episiotomy on indication has been introduced in many obstetrics units in the Nordic countries, but the evidence for this intervention is extremely limited [15]. A non-randomized study from Sweden showed that a multifaceted intervention consisting of spontaneous pushing, birth positions with flexibility in the sacroiliac joints, and a two-step head-to-body delivery significantly reduced second degree tears, but these results have not yet been reproduced [3]. There is a need for new interventions to prevent severe perineal tears, and one way to approach such measures is epidemiologic research regarding risk factors for perineal and high vaginal tears.

Instrumental delivery [4, 5], protracted second stage of labor [5, 16], birth weight greater than 4 kg [16], and fetal occipito-posterior presentation [4, 16] have been shown to be independent risk factors for OASI in several retrospective studies. Retrospective studies have generally focused on OASI, whereas second-degree tears have almost exclusively been reserved for prospective observational studies. Only six articles based on prospective observation studies of OASI and/or other perineal tears were identified in an extensive PubMed search [1, 2, 17–20], and only two of these articles included second-degree perineal tears [1, 2].

The aim of this study was to estimate the incidence of second-degree perineal tears, OASI (defined as any third or fourth degree perineal tear), and high vaginal tears in primiparous women, and to examine how sociodemographic and pregnancy characteristics, hereditary factors, obstetric management and the delivery process are associated with the incidence of these tears.

## Methods

### Study design and population

We conducted a prospective cohort study in the Region Örebro County, Sweden, named the **P**elvic Fl**o**or In **Pr**egnancy **A**nd **C**hildbir**t**h (POPRACT) study. All eligible nulliparous women registering for maternity health care in early pregnancy between 1 October 2014 and 1 October 2017 were informed about the study and asked if they wanted to participate by the midwife in charge. Antenatal care is free of charge in Sweden, and almost all women attend maternity health care. Exclusion criteria were first visit at maternity health care after 15 weeks + 6 days of gestation or insufficient knowledge of the Swedish language to complete the questionnaires used in the study. Participants were asked to complete web-based questionnaires on four occasions: at entry into the study in early pregnancy, at 36 weeks of gestation, at 8 weeks postpartum, and at 1 year postpartum. Patient-reported data were managed in the cloud-based tool esMaker 3.0 (Entergate AB, Sweden) in accordance with the General Data Protection Regulation of the European Union. The questionnaires included items on general health, socioeconomic status, heredity of pelvic floor dysfunction and connective tissue deficiency, self-reported pelvic floor dysfunction [21, 22] quality of life related to pelvic floor dysfunction [23] and sexual function related to pelvic floor dysfunction [24], see Additional file 1.

### Study size

The present study is a first report from the POPRACT study that aims at studying risk factors for perineal and vaginal birth trauma and subsequent impact on pelvic floor dysfunction including Quality of Life and sexual function. Given the multiple outcomes with unknown incidence, the required sample size for the whole study was difficult to estimate precisely. Inclusion was terminated after three years when slightly more than 1000 women had been included which was judged to be sufficient for detecting risk factors for most outcomes although perhaps not for rare risk factors. For perineal tears, given the incidence reported in the literature for OASI of 5.1–8.3% [1, 2, 4, 5] and significantly higher for second degree tears, a study population of 1000 women was judged considered to be sufficient to identify risk factors of clinical importance.

### Exposure measures

The following patient-reported data from the first and second questionnaires (i.e. before delivery) were analyzed as potential risk factors for perineal tears and vaginal tear: level of education, heredity of pelvic floor disorders and/or connective tissue deficiency, symptoms of stress urinary incontinence, and symptoms of pelvic organ prolapse. Heredity of pelvic floor disease was defined as mother or sister having undergone surgery due to pelvic organ prolapse, urinary incontinence, inguinal hernia, or varicose veins. Stress urinary incontinence was defined as reporting urine leakage “often” or “sometimes” during physical strain. Symptoms of pelvic organ prolapse was defined as responding “often” or “sometimes” to the question about the sensation of vaginal bulging. Patient-reported data about symptoms of pelvic floor dysfunction and quality of life and sexual function related to pelvic floor dysfunction after delivery will be presented in separate scientific publications.

Participating women had their delivery at either of the two delivery wards in Region Örebro County, which are located at Örebro University Hospital and at Karlskoga Hospital. Delivery was assisted by a midwife under ordinary circumstances or by an obstetrician in case of instrumental delivery. Diagnosis of first- and second-degree perineal tears was made by a midwife. In cases of suspected third- or fourth-degree perineal tear or a high vaginal tears, an obstetrician was consulted for an assessment and suturing. After delivery, vaginal examination, and suturing if necessary, the midwife (in co-operation with the obstetrician when needed) completed a study protocol containing specific questions about delivery characteristics, perineal and vaginal tears, and suturing. The part of the protocol regarding perineal tears and suturing has been validated in a previous study [25]. The extent of the perineal or vaginal tear was judged by eye by the midwife or obstetrician, and were classified according to the Royal College of Obstetricians and Gynaecologists classification of perineal tears [26] and the ICD-10 classification of high vaginal tear; that is, a vaginal tear extending above the distal third of the vagina [27]. These classifications are used in the current obstetric record system and are described in the above-mentioned protocol. In case of episiotomy the perineal tear was classified as second-degree at minimum. In women who had both episiotomy and a perineal tear of third or fourth degree, the classification of perineal tear remained unchanged. In order to avoid confounding the incidence and risk factor analysis of perineal tears, women having an episiotomy were excluded from the these analyses. Information regarding oxytocin augmentation during active second stage of labor, use of episiotomy, manual perineal protection, and application of fetal scalp electrode was retrieved from the mentioned study protocol. Data concerning BMI at maternity health care registration in early pregancy, smoking at maternity health care registration in early pregnancy, maternal age at delivery, gestational age at birth, whether delivery started spontaneously or was induced, administration of epidural analgesia, duration of active second stage of labor, maternal position at birth, mode of delivery, fetal presentation, fetal birth weight, and fetal head circumference were extracted from the obstetric record system (Obstetrix version 2.16.0.200, Cerner Corporation, Sweden) using an accessory program (Obstetrix Förlossningsliggare version 2.16.0.200, Cerner Corporation, Sweden). According to the midwife-in-chief at the participating delivery wards, the practice at the time of the study was to define active second stage of labor as active pushing. Variables were categorized as follows: age was categorized into ≤25 years and > 25 years; BMI into ≤25 kg/m^2^, 25.1–30 kg/m^2^ and > 30 kg/m^2^; gestational age at delivery into preterm (< 37 + 0), term (37 + 0–42 + 0), and postterm (> 42 + 0); duration of active second stage of labor into ≤15 min, 16–60 min, and > 60 min; mode of delivery into spontaneous and vacuum extraction; fetal presentation into occiput anterior and occiput posterior; fetal weight into ≤4000 g and > 4000 g; and fetal head circumference into ≤35 cm and > 35 cm. Maternal position at birth was categorized into 1) flexible sacrum positions, including squatting, kneeling and lateral; and 2) positions with reduced sacrum flexibility, including lithotomy, supine and sitting.

### Outcome measures

The primary outcome measure was perineal tear, which was divided into three groups: 1) intact perineum or first degree tear (defined as the reference category), 2) second-degree tear, and 3) third- or fourth-degree tear, i.e. OASI. Vaginal tears, were categorized into two groups: 1) no or low vaginal tear (the reference category) and 2) high vaginal tear.

### Statistical analyses

Relationships between potential risk factors and different degrees of perineal and vaginal tears were evaluated using unadjusted and adjusted multivarable regression models. Multinomial logistic regression was used for perineal tears, and logistic regression was used for vaginal tears. In the multivariate models for perineal tear, all potential risk factors were entered in the model and mutually adjusted for except heredity of pelvic floor dysfunction and/or connective tissue deficiency, stress urinary incontinence, episiotomy, whether hand or arm was the presenting part, and fetal head > 35 cm. In the case of vaginal tear, all risk factors except stress urinary incontinence and fetal weight > 4000 g were entered in the adjusted model. Assessment of potential multicollinearity among risk factors showed no collinearity issues; all variance inflation factors were < 1.6. An interaction between fetal weight and delivery mode on the risk of perineal tear was examined using interaction tests.

An additional risk factor analysis including women having an episiotomy was performed. In this analysis, episiotomy was evaluated as a risk factor of OASI, but was not included in the final analysis due to too few women having the combination of episiotomy and OASI.

Differences between vaginally delivered women with and without a registered study-specific delivery protocol were compared using a t-test in the case of supposed parametric continuous variables, the Wilcoxon rank-sum test in the case of supposed non-parametric continuous variables, and a chi-squared test in the case of categorical variables. Data were analyzed using version Stata/SE V13 (StataCorp LP, College Station, TX).

## Results

**Fig. 1:**
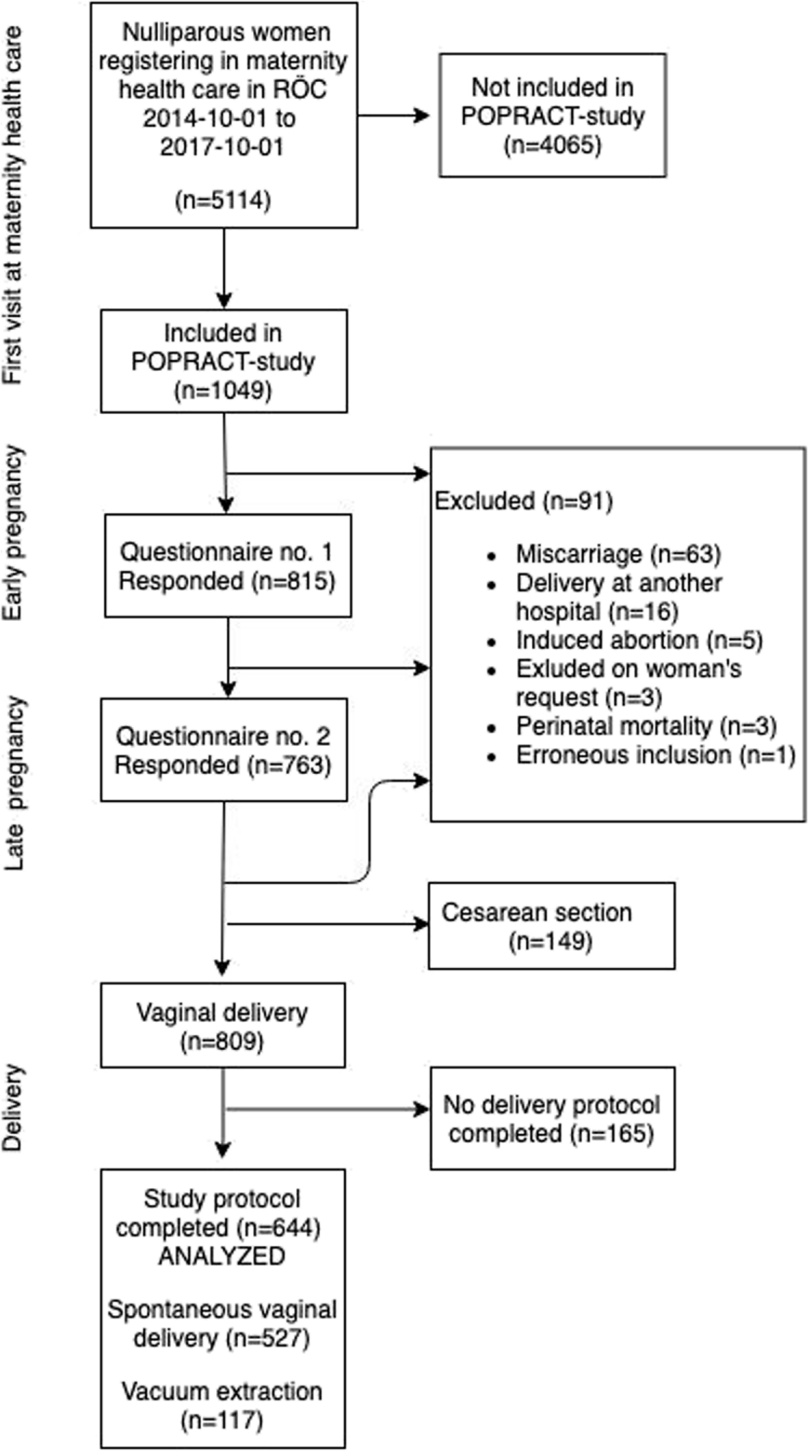
Flow chart illustrating the inclusion of the study sample. RÖC, Region Örebro County; POPRACT study, Pelvic Floor In Pregnancy And Childbirth study

Figure 1 presents the inclusion of the study sample. A total of 1049 women were included in the POPRACT study. Of the study population remaining after exclusion, 809 women had a vaginal delivery. Delivery was documented in the dedicated study protocol for 644 of these women, who thus constituted the present study sample. The analysis of risk factors in relation to perineal and vaginal tears included 443 and 421 women, respectively, after excluding women with missing data in relevant variables.

Baseline and obstetric and baseline characteristics of the study sample are shown in Table 1 and Table 2, respectively. The sample had a mean (± SD) age of 28.7 ± 3.7 years (range: 18–41 years), BMI of 24.5 ± 4.4 kg/m^2^ (16.4–44.0 kg/m^2^), gestational age at birth of 40 weeks + 1 day ±1 week + 3 days (34 weeks + 1 day – 42 weeks + 5 days), fetal birth weight of 3513 ± 472 g (1730–5140 g), and fetal head circumference of 34.8 ± 1.5 cm (28.0–38.5 cm). Smoking, symptoms of pelvic organ prolapse during late pregnancy, and lack of manual perineal protection were considered as potential risk factors but were excluded from the analysis of risk factors presented below due to too few exposed women. No statistically significant differences were found between the women whose data were collected according to study-specific delivery protocol registered (*n* = 644) and those excluded due to missing study protocol (*n* = 165), except regarding use of epidural analgesia and duration of active second stage of labor. In the excluded group, epidural use was lower (38.2%) and the mean duration of active second stage of labor was longer (48.7 ± 35.4 min; range: 1–189 min).

**Table 1:** Baseline characteristics of the study population

	n (%)
**Age**	
≤ 25 years	114 (17.7)
26–30 years	347 (53.9)
31–35 years	155 (24.1)
> 35 years	28 (4.4)
Missing	0
**BMI**	
≤ 25 kg/m^2^	405 (64.5)
25.1–30 kg/m^2^	155 (24.7)
> 30 kg/m^2^	68 (10.8)
Missing	16
**Smoking**	
Yes	19 (3.0)
No	605 (97.0)
Missing	20
**Education**	
9–< 12 years	8 (1.5)
12 years	181 (33.2)
University	357 (65.4)
Missing	98
**Heredity**^**a**^	
Yes	70 (14.4)
No	415 (85.6)
Missing	159
**SUI during late pregnancy**	
Yes	116 (22.2)
No	406 (77.8)
Missing	122
**Symptoms of POP during late pregnancy**	
Yes	22 (4.2)
No	501 (95.8)
Missing	121

Baseline characteristics of the study population. Women where information is missing are not included in the percentage. ^a^Heredity of pelvic floor dysfunction and/or connective tissue deficiency. *BMI*, body mass index; *POP*, pelvic organ prolapse; *SUI*, stress urinary incontinence

**Table 2:** Obstetric characteristics of the study population

	n (%)
**Gestational age at birth**	
Preterm (<37w)	21 (3.3)
Term (37–42w)	552 (85.7)
Postterm (>42w)	60 (9.3)
Missing	11
**Delivery start**	
Spontaneous	512 (79.6)
Induction	131 (20.4)
Missing	1
**Epidural analgesia**	
No	314 (48.8)
Yes	330 (51.2)
Missing	0
**Oxytocin stimulation**	
No	306 (48.5)
Yes	325 (51.5)
Missing	12
**Duration of active 2nd stage**	
≤ 15 min	124 (19.9)
16–60 min	346 (55.5)
> 60 min	154 (24.7)
Missing	20
**Episiotomy**	
No	579 (91.3)
Yes	55 (8.7)
Missing	9
**Maternal position at birth**	
Lithotomy	353 (55.6)
Squatting	1 (0.16)
Kneeling	11 (1.7)
Supine	18 (2.8)
Lateral	98 (15.2)
Sitting	153 (23.8)
Standing	1 (0.16)
Missing	9
**Mode of delivery**	
Spontaneous	527 (81.8)
Instrumental	117 (18.2)
Missing	0
**Manual perineal protection**	
None	8 (1.3)
Fetal head support only	30 (4.8)
Perineal head support only	82 (13.0)
Combined support	409 (65.0)
Unspecified support	100 (15.9)
Missing	15
**Fetal scalp electrode**	
No	279 (44.2)
Yes	352 (55.8)
Missing	12
**Fetal presentation**	
Occiput anterior	611 (96.4)
Occiput posterior	23 (3.6)
Breech	0
Missing	10
**Fetal birth weight**	
≤ 4000 g	541 (84.1)
> 4000 g	102 (15.9)
Missing	1
**Fetal head circumference**	
≤ 35 cm	278 (43.4)
> 35 cm	363 (56.6)
Missing	3
**Number of births**	
Singleton	641 (99.7)
Twins	2 (0.3)
Missing	1

Obstetric characteristics of the study population. Women where information is missing are not included in the percentage

### Incidence of perineal, vaginal, and other vulvar tears

Table 3 presents the incidence of vaginal, perineal, and other vulvar tears. Almost half of the women (47.6%) contracted any labial tear requiring suturing. Anterior tears close to the clitoris or urethra were less common than labial tears, affecting 15.3% of the sample. Only 14.9% of women avoided any vaginal tear. The vast majority (71.1%) of women with vaginal tear had a low tear, whereas 14.0% contracted a high vaginal tear. About one third (33.7%) of these women had an intact perineum, while the remaining two thirds had some degree of perineal tear. Second-degree tears constituted the majority of tears (40.6%). The incidences of third-degree tears of class A, B, and C were 4.1, 1.1, and 2.1% respectively. Only two women (0.35%) contracted a fourth-degree perineal tear. The incidence of perineal tear in women having an episiotomy or with no information regarding episiotomy, respectively, is presented separately.

**Table 3:** Distribution of vaginal, perineal, and other vulvar tear

Labial tears^**a**^ (*n* = 644)	n (%)
None	329 (52.4)
Yes	299 (47.6)
Missing	16
**Anterior tears**^**b**^ (*n* = 644)	n (%)
None	511 (84.7)
Yes	92 (15.3)
Missing	41
**Vaginal tear** (*n* = 644)	n (%)
None	91 (14.9)
Low^c^	433 (71.1)
High^d^	85 (14.0)
Missing	35
**Degree of perineal tear** (*n* = 580)	n (%)
None	191 (33.7)
First-degree	103 (18.2)
Second-degree	230 (40.6)
Third-degree (A)	23 (4.1)
Third-degree (B)	6 (1.1)
Third-degree (C)	12 (2.1)
Fourth-degree	2 (0.35)
Missing	13
**Degree of perineal tear, women with episiotomy** (*n* = 55)	n (%)
Episiotomy without OASI	51 (94.4)
Episiotomy and third-degree (A)	1 (1.9)
Episiotomy and third-degree (B)	2 (3.7)
Missing	1
**Degree of perineal tear, women with missing information regarding episiotomy** (*n* = 9)	n (%)
None	3 (50)
First-degree	1 (16.7)
Second-degree	2 (33.3)
Missing	3

Distribution of vaginal, perineal, and other vulvar tear. An individual woman may have labial, anterior, vaginal and perineal tear concomitantly and thus be part of several tear groups. Missing information is due to incomplete information in the delivery protocols and is not included in the percentage. ^a^labial tears requiring suturing; ^b^anterior tears close to clitoris or urethra, not related to female genital mutilation; ^c^vaginal tear where only the distal third of vagina is engaged; ^d^vaginal tear more extensive than the distal third of vagina. *OASI*,obstetric anal sphincter injury

### Odds ratios for the risk factors of second-degree perineal tear and OASI

Table 4 presents the unadjusted and adjusted odds ratios for second-degree perineal tear and for OASI, respectively. Women with second-degree perineal tear were more likely to be older than 25 years, to have a post-term delivery, to be exposed to oxytocin augmentation, to have an active second stage shorter or equal to 15 min, to have delivery assisted by vacuum extraction, to have fetal heart beat monitored by scalp electrode, and to have a child heavier than 4000 g or with a head circumference exceeding 35 cm, compared to women who did not have a tear or had a tear of first degree (the reference). After adjustment, post-term delivery, vacuum extraction, and fetal weight exceeding 4000 g remained as risk factors significantly increasing the risk of second-degree perineal tear. In the adjusted model, maternal birth positions with reduced sacrum flexibility, significantly decreased the risk of second-degree perineal tear, despite not being significant in the unadjusted model. Women with OASI were more likely to use epidural analgesia, to have delivery assisted by vacuum extraction, to have fetal heart beat monitored by scalp electrode, and to have a child heavier than 4000 g or with a head circumference exceeding 35 cm, compared to the reference. After adjustment, vacuum extraction and fetal weight > 4000 g remained as risk factors significantly increasing the risk of OASI. In the analysis including women having an episiotomy, age was an independent risk factor of second-degree perineal tear, see additional file 2. Otherwise no significant differences were found.

**Table 4:** Unadjusted and adjusted odds ratios for risk factors for perineal tear

*n* = 443	2nd degree (*n* = 182)		OASI (*n* = 31)	
	OR (95% CI)	aOR (95% CI)	OR (95% CI)	aOR (95% CI)
**Age**				
≤ 25 years	Reference	Reference	Reference	Reference
> 25 years	1.78 (1.05, 3.04)*	1.62 (0.90, 2.93)	1.41 (0.51, 3.86)	1.36 (0.40, 4.56)
**BMI**				
≤ 25 kg/m^2^	Reference	Reference	Reference	Reference
25.1–30 kg/m^2^	1.02 (0.64, 1.61)	1.11 (0.68, 1.81)	1.21 (0.52, 2.8)	1.04 (0.41, 4.56)
> 30 kg/m^2^	1.19 (0.63, 2.22)	1.22 (0.61, 2.41)	0.63 (0.14, 2.85)	0.38 (0.07, 1.99)
**Education**				
9to < 12 years	0.3 (0.03, 2.69)	0.35 (0.04, 3.47)	3.32 (0.57, 19.2)	4.01 (0.54, 29.8)
12 years	0.86 (0.57, 1.3)	0.93 (0.59, 1.46)	0.58 (0.24, 1.42)	0.56 (0.20, 1.55)
University	Reference	Reference	Reference	Reference
**Heredity**^**a**^ (*n* = 397)				
No	Reference	NE	Reference	NE
Yes	1.38 (0.77, 2.46)	NE	1.63 (0.57, 4.7)	NE
**SUI in late pregnancy**				
No	Reference	NE	Reference	NE
Yes	0.69 (0.42, 1.12)	NE	1.59 (0.7, 3.63)	NE
**GA at birth**				
Preterm/term	Reference	Reference	Reference	Reference
Postterm	2.23 (1.11, 4.47)*	2.44 (1.03, 5.77)*	2.29 (0.7, 7.45)	1.48 (0.34, 6.50)
**Delivery start**				
Spontaneous	Reference	Reference	Reference	Reference
Induction	1.16 (0.7, 1.93)	0.73 (0.38, 1.40)	1.76 (0.73, 4.22)	1.31 (0.43, 4.00)
**Epidural analgesia**				
No	Reference	Reference	Reference	Reference
Yes	1.20 (0.81, 1.77)	0.97 (0.63, 1.5)	2.41 (1.09, 5.35)*	1.62 (0.68, 3.87)
**Oxytocin stimulation**				
No	Reference	Reference	Reference	Reference
Yes	1.53 (1.03, 2.26)*	1.18 (0.74, 1.9)	1.9 (0.89, 4.06)	0.85 (0.34, 2.13)
**Duration of active 2nd stage**				
≤ 15 min	0.66 (0.39, 1.14)*	0.69 (0.39, 1.21)	1.08 (0.43, 2.76)	1.22 (0.44, 3.4)
16–60 min	Reference	Reference	Reference	Reference
> 60 min	1.09 (0.68, 1.74)	0.99 (0.6, 1.62)	0.84 (0.32, 2.23)	0.59 (0.21, 1.72)
**Maternal position at birth**				
Flexible				
sacrum	Reference	Reference	Reference	Reference
positions				
Reduced	0.94 (0.59, 1.49)	0.53 (0.32, 0.90)*	1.07 (0.42, 2,75)	0.63 (0.21, 1.85)
sacrum				
flexibility				
**Mode of delivery**				
Spontaneous	Reference	Reference	Reference	Reference
Vacuum				
extraction	2.37 (1.29, 4.34)*	2.41 (1.24, 4.68)*	3.86 (1.52, 9.8)*	3.91 (1.32, 11.6)*
**Fetal scalp electrode**				
No	Reference	Reference	Reference	Reference
Yes	1.28 (0.87, 1.9)*	1.13 (0.73, 1.77)	3.03 (1.3, 7.05)*	2.55 (0.98, 6.61)
**Fetal presentation**				
Occiput anterior	Reference	Reference	Reference	Reference
Occiput posterior	1.27 (0.44, 3.7)	1.38 (0.45, 4.21)	2.2 (0.44, 11.08)	3.22 (0.53, 19.5)
**Hand or arm presenting fetal part**				
No	Reference	NE	Reference	NE
Yes	1.05 (0.57, 1.91)	NE	0.81 (0.23, 2.83)	NE
**Fetal weight**				
≤ 4000 g	Reference	Reference	Reference	Reference
> 4000 g	2.46 (1.35, 4.49)*	2.22 (1.17, 4.22)*	6.11 (2.55, 14.6)*	6.02 (2.32, 15.6)*
**Fetal head circumference**				
≤ 35 cm	Reference	NE	Reference	NE
> 35 cm	1.87 (1.26, 2.77)*	NE	3.94 (1.63, 9.51)*	NE

Unadjusted and adjusted odds ratios for risk factors for perineal tear using multinomial logistic regression. The group of women with second-degree perineal tear and OASI were compared with women with no or first-degree perineal tear. Women having an episiotomy were excluded from the analysis. Sample size for the unadjusted OR for heredity, SUI, hand or arm presenting fetal part and fetal head circumference was based on n = 397, *n* = 415, *n* = 441 and *n* = 442, respectively. ^a^Heredity of pelvic floor dysfunction and/or connective tissue deficiency; *Significant at level *p* < 0.05. *aOR* adjusted odds ratio, *BMI* body mass index, *CI* confidence interval, *GA* gestational age, *NE* not estimated, *OASI* obstetric anal sphincter injury, *OR* odds ratio, *SUI* stress urinary incontinence

### Odds ratios for high vaginal tear

Table 5 shows the unadjusted and adjusted odds ratios for high vaginal tear. Women with a high vaginal tear were more likely to report heredity of pelvic floor dysfunction and/or connective tissue deficiency, to have induced labor, to deliver a baby whose hand or arm was the presenting fetal part, and to deliver a baby whose head circumference exceeded 35 cm, compared to referent women with no or low vaginal tear. After adjustment, heredity of pelvic floor dysfunction and/or connective tissue deficiency, induced labor, and fetal head circumference > 35 cm remained as risk factors, significantly increasing the risk of high vaginal tear. In the adjusted model, vacuum extraction significantly increased the risk of high vaginal tear, whereas augmentation of oxytocin significantly reduced the risk of high vaginal tear, despite none of them being significantly associated with high vaginal tear in the unadjusted model.

**Table 5:** Unadjusted and adjusted odds ratio for the risk of high vaginal tear

*n* = 421	High vaginal tear (*n* = 55)	
	OR (95% CI)	aOR (95% CI)
**Age**		
≤ 25 years	Reference	Reference
> 25 years	2.20 (0.84, 5.73)	2.36 (0.77, 7.26)
**BMI**		
≤ 25 kg/m^2^	Reference	Reference
25.1–30 kg/m^2^	1.12 (0.57, 2.22)	1.15 (0.54, 2.47)
> 30 kg/m^2^	0.97 (0.39, 2.45)	0.85 (0.3, 2.37)
**Education**		
9 to < 12 years	1.48 (0.16, 13.53)	5.83 (0.45, 75.33)
12 years	0.64 (0.33, 1.24)	0.79 (0.38, 1.64)
University	Reference	Reference
**Heredity**^**a**^		
No	Reference	Reference
Yes	2.21 (1.12, 4.35)*	2.32 (1.09, 4.97)*
**SUI in late pregnancy**		
No	Reference	NE
Yes	0.81 (0.38, 1.74)	NE
**GA at birth**		
Preterm and term	Reference	Reference
Postterm	2.04 (0.92, 4.55)	0.69 (0.23, 2.05)
**Delivery start**		
Spontaneous	Reference	Reference
Induction	2.64 (1.4, 4.95)*	3.16 (1.31, 7.62)*
**Epidural analgesia**		
No	Reference	Reference
Yes	0.93 (0.53, 1.63)	0.78 (0.4, 1.5)
**Oxytocin stimulation**		
No	Reference	Reference
Yes	0.83 (0.47, 1.47)	0.41 (0.2, 0.84)*
**Duration of active 2nd stage**		
≤ 15 min	0.65 (0.27, 1.53)	0.71 (0.29, 1.76)
16–60 min	Reference	Reference
> 60 min	1.05 (0.54, 2.03)	0.97 (0.46, 2.02)
**Episiotomy**		
No	Reference	Reference
Yes	1.54 (0.6, 3.91)	1.01 (0.34, 3.05)
**Maternal position at birth**		
Flexible sacrum positions	Reference	Reference
Reduced sacrum flexibility	1.30 (0.61, 2.77)	1.08 (0.46, 2.53)
**Mode of delivery**		
Spontaneous	Reference	Reference
Vacuum extraction	1.55 (0.78, 3.06)	2.53 (1.07, 5.98)*
**Fetal scalp electrode**		
No	Reference	Reference
Yes	1.59 (0.88, 2.85)	1.71 (0.85, 3.42)
**Fetal presentation**		
Occiput anterior	Reference	Reference
Occiput posterior	0.43 (0.06, 3.35)	0.47 (0.04, 5.07)
**Hand or arm presenting fetal part**		
No	Reference	Reference
Yes	2.16 (1.03, 4.53)*	2.27 (0.99, 5.24)
**Fetal weight**		
≤ 4000 g	Reference	NE
> 4000 g	1.37 (0.65, 2.9)	NE
**Fetal head circumference**		
≤ 35 cm	Reference	Reference
> 35 cm	2.71 (1.41, 5.22)*	3.07 (1.5, 6.3)*

Unadjusted and adjusted odds ratio for the risk of high vaginal tear using logistic regression. The group of women with high vaginal tear was compared with women with none or low vaginal tear. Sample size for the unadjusted OR for SUI was based on *n* = 392. ^a^Heredity of pelvic floor dysfunction and/or connective tissue deficiency; *Significant at level *p* < 0.05. *aOR* adjusted odds ratio, *BMI* body mass index, *CI* confidence interval, *GA* gestational age, *NE* not estimated, *OR* odds ratio, *SUI* stress urinary incontinence

### Odds ratios for the combined effect of delivery mode and fetal weight on the risk of perineal tear

Table 6 shows the odds ratios, before and after adjustment, for second-degree perineal tear and OASI, in four different combinations of two risk factors - vacuum extraction and fetal weight: 1) women with spontaneous delivery of a child weighing < 4000 g, 2) women with spontaneous delivery of a child weighing ≥4000 g, 3) women with vacuum-assisted delivery of a child weighing < 4000 g, and 4) women with vacuum-assisted delivery of a child weighing ≥4000 g. Subgroup 4, in which the two major risk factors were combined, had adjusted ORs for second-degree tear and OASI of 4.8 (95% CI: 1.20, 19.3) and 12.7 (95% CI: 1.65, 97.7), respectively, and the interaction terms for second-degree perineal tear and for OASI were 0.89 (95% CI: 0.17, 4.66) and 0.30 (95% CI: 0.03, 3.16), respectively, meaning that there was no significant interaction between vacuum extraction and fetal birthweight above 4000 g (data not shown).

**Table 6:** Unadjusted and adjusted odds ratios for the risk of second-degree perineal tear and obstetric anal sphincter injury by delivery mode and fetal weight

*n* = 443	Second-degree perineal tear (*n* = 182)			Obstetric anal sphincter injury (*n* = 31)		
	Incidence (n)	OR (95% CI)	aOR (95% CI)	Incidence (n)	OR (95% CI)	aOR (95% CI)
Spontaneous delivery and fetal weight < 4000 g (*n* = 335)	126	Reference	Reference	14	Reference	Reference
Spontaneous delivery and fetal weight ≥ 4000 g (*n* = 49)	24	2.32 (1.19, 4.54)*	2.22 (1.1, 4.51)*	9	7.83 (2.94, 20.9)	7.7 (2.71, 21.8)*
Vacuum extraction and fetal weight < 4000 g (*n* = 45)	23	2.22 (1.13, 4.37)*	2.41 (1.16, 5.02)*	6	5.22 (1.77, 15.4)*	5.52 (1.62, 18.8)*
Vacuum extraction and fetal weight ≥ 4000 g (*n* = 17)	9	4.64 (1.23, 17.5)*	4.8 (1.20, 19.3)*	2	9.29 (1.43, 60.2)*	12.7 (1.65, 97.7)*

Unadjusted and adjusted odds ratios for the risk of second-degree perineal tear and obstetric anal sphincter injury by delivery mode (spontaneous or vacuum extraction) and fetal weight (< 4000 g or ≥ 4000 g). *Significant at level *p* < 0.05. *aOR* adjusted odds ratio, *CI* confidence interval, *OR* odds ratio

## Discussion

In this prospective study of primiparous women, the incidences of second-degree perineal tear, OASI, and high vaginal tear were 40.6, 7.4, and 14.0% respectively. Vacuum extraction and fetal weight above 4000 g were independent risk factors for both second-degree perineal tear and OASI. Post-term delivery significantly increased the risk for second-degree perineal tear, and, surprisingly, maternal birth positions with reduced sacrum flexibility significantly decreased the risk of second-degree perineal tear, whereas none of them were significantly associated with OASI. Heredity of pelvic floor dysfunction and/or connective tissue deficiency, induced labor, vacuum extraction and fetal head circumference exceeding 35 cm were independent risk factors for high vaginal tear, whereas oxytocin augmentation, unexpectedly, appeared to reduce the risk of high vaginal tear.

To our knowledge, this is one of very few observational studies of perineal tears that include tears of second degree. An extensive PubMed search identified only two observational studies reporting the incidence of second-degree perineal tear [1, 2] and only one of these separately analyzed risk factors for second-degree tears [1]. As in the present study, Samuelsson et al. found high infant weight to be an independent risk factor for both second-degree tears and OASI, but in their study vacuum extraction was not an independent risk factor for either degree of tear. We did not find that prolonged active phase of second stage of labor led to any increased risk for either OASI or second-degree tears, whereas Samuelsson et al. found that pushing time < 30 min decreased the risk of both [1, 17]. An imprecise definition of the active phase of second stage of labor in the present study might partly explain the difference in the results; a review of the obstetric record of all women with active second stage of labor exceeding 120 min revealed that in about half of those cases, the midwife entered the time when the woman felt urge to push whereas the active pushing appeared to start later, which may have obscured an effect of the length of active pushing in our study.

We found an incidence of second-degree tears of 40.6%, which is similar to the findings of Samuelsson et al. [1] but considerably lower than the incidence of 78.3% reported in the control group of an interventional study by Edqvist et al. [3]. Since the latter study was also conducted in a Swedish context and published as recently as 2017, explanations other than a true difference in the incidence due to diverging obstetric practice must be sought. Rather, diverging definitions of second-degree tears could explain the difference. Our study and Samuelsson et al. [1] used the RCOG definitions of perineal tears [26], whereas Edqvist et al. classified vaginal tears with a depth > 0.5 cm as second-degree tears [3]. Unexpectedly, we found positions with reduced sacrum flexibility to be protective of second-degree perineal tear. This contradicts the finding of Edqvist et al. [3], whose intervention including flexible sacrum positions significantly reduced second-degree perineal tears. However, the evidence supporting any birth position to be superior to another in preventing perineal tears is limited [28, 29].

The incidence of OASI of 7.4% in the present study is among the highest reported to our knowledge. The majority of previous studies have reported a lower incidence of OASI in primiparous women, ranging 5.1–6.7% [1, 2, 4], although one study found a higher incidence of 8.3% [5]. Obstetric management may partly explain our high incidence, such as the comparably frequent use of instrumental delivery of 18.2% in our study. The accuracy of incidence data must also be addressed when comparing studies. A validity study reported that one of four hospital discharges associated with OASI were undercoded [30], thus questioning the results of retrospective studies based on discharge codes. Finally, the incidence of OASI in epidemiologic studies, including ours, almost exclusively relies on clinical diagnosis of OASI. Clinical diagnosis of OASI is known to be difficult, generally underestimating the incidence compared to endoanal sonography [31].

The use of episiotomy poses a challenge when studying second-degree perineal tears since episiotomy technically is a second-degree tear, however iatrogenic. A woman having an episiotomy must be considered to have a second-degree perineal tear at a minimum since an episiotomy appears to be associated with at least the same risk of complications and chronic ailments as a spontaneous second-degree tear [14]. However, including women having an episiotomy when studying incidence and risk factors of second-degree perineal tears exaggerate the incidence of the latter and confound the analysis of risk factors. Consequently we excluded the women with episiotomy when calculating incidence and analyzing risk factor of perineal tears. Although the exclusion may be seen to reduce the generalizability of our results, the analysis including the women with episiotomy showed similar results to our main analysis.

Obstetric risk factors for perineal tears are often interrelated, as is the case for the two largest risk factors identified in this study: birth weight > 4000 g and vacuum extraction. This was the rationale for the stratification of subgroups according to these risk factors (Table 6). The odds of OASI in the subgroup with the two major risk factors combined was markedly high; more than tenfold higher than the reference category, even though there was no evidence of positive effect modification and confidence intervals were wide.

High vaginal tear was fairly common in our study, affecting 14.0% of women. Our review of the literature found only two studies specifically reporting the incidence of vaginal tears, ranging 7.8–35.1%, irrespective of parity [32, 33]. However, none of the studies reported the extension of vaginal tears, albeit one of the studies used a detailed protocol including information about the extension of vaginal tears [33]. We found no study exploring the risk factors of vaginal tears.

Vaginal sidewall tears might be an independent risk factor for levator ani avulsion [10], and hence could be a marker for increased future risk of pelvic floor dysfunction. Interestingly enough, we found that heredity of pelvic floor dysfunction and/or connective tissue deficiency was a risk factor for high vaginal tear. One might speculate that a genetic connective tissue deficiency resulting in an increased risk of levator ani avulsion is the link, which explains the finding above. Vacuum extraction has earlier been associated with increased risk of levator ani avulsion [34]. This possibly supports the present finding of vacuum extraction being a risk factor of high vaginal tear, given the association between vaginal sidewall tears and levator ani avulsion decribed above. The associations found between high vaginal tear and induction of labor and oxytocin augmentation respectively, we consider should be interpreted with caution.

Strengths of this study is the prospective data collection and the assessment of a wide range of risk factors. In the present study we used a validated protocol for documentation of perineal tears, which we have previously shown to deliver more comprehensive information about perineal tears than the most common obstetric record system in Sweden [25].

Although we examined a range of variables, there a several potential risk factors and protective factors not being considered in the present study. For example, we could not evaluate the application of warm compresses to the perineum during delivery or the use of antenatal perineal massage as protective factors, because these variables were not included in the study protocol or in any template of the obstetric record system.

The sample size of this prospective study (489 and 426 women included in the regression models of perineal tears and vaginal tears respectively) is smaller than in most retrospective studies in the field, which constitutes a limitation of the study. The limited sample size confers a risk of type II errors, and may partly explain why some previously described risk factors did not show the association. Our study was exploratory, and the associations suggested in our study may therefore be important to be examined with a study with greater sample size and higher previsions in data.

Data collected in a context of daily clinical practice may have led to imprecise recording of some variables. As discussed elsewhere, the definition of active second stage of labor varied, and the eye-assessment of high vaginal rupture cannot be claimed to be exact. Such misclassification of variables might have resulted in spurious significant associations or in underestimation of associations to a degree. On the other hand, the results from a study performed in a clinical context may be transferable to everyday practice to a higher extent, than the results from a controlled clinical trial.

## Conclusions

The present study corroborates previous findings of vacuum extraction and fetal weight exceeding 4000 g as risk factors of OASI. We found that vacuum extraction is a risk factor for second-degree tear, and vacuum extraction, fetal head circumference exceeding 35 cm and heredity of pelvic floor dysfunction and/or connective tissue deficiency were associated with increased risk of high vaginal tears. These findings have not been documented previously. Sociodemographic factors did not appear to affect the risk for neither tear. Important findings were the high incidences of second-degree perineal tear and high vaginal tear, which have not been sufficiently examined before. Our results should be confirmed by additional studies.

## Supplementary Information

**Additional file 1.**

**Additional file 2.**

## Data Availability

The datasets used and analyzed during the current study are available from the corresponding author on reasonable request.

## Abbreviations

OASI: Obstetric anal sphincter injury
POPRACT: Pelvic Floor In Pregnancy and Childbirth study
