# Supplementary material for: Risk factors for perineal and vaginal tears in primiparous women – the prospective POPRACT-cohort study
Source: BMC Pregnancy Childbirth. 2020 Dec 2;20:749. doi: 10.1186/s12884-020-03447-0 (PMC7709229; doi:10.1186/s12884-020-03447-0)
Supplement: Supplementary file 1 — Additional file 1. [file 12884_2020_3447_MOESM1_ESM.pdf]

QUESTIONNAIRE administered: 1) when registering at the maternity health care, 2) in late pregnancy, 3) at 8–10 weeks postpartum, 4) at 1 year postpartum

To women participating in a scientific study concerning pelvic floor dysfunction in connection with pregnancy and first delivery

---

Date of completion __________________

1. What is your weight today? ________
2. How tall are you? _________
3. What is the highest level of education that you have completed?
    - o Nine-year compulsory school or equivalent
    - o Upper secondary school or equivalent
    - o University or college

Hereditary factors (when registering at the maternity health care only)

4. Has your mother or sister had an operation due to prolapse?
    - o Yes
    - o No
    - o I do not know
5. Has your mother or sister had an operation due to urinary incontinence?
    - o Yes
    - o No
    - o I do not know
6. Has your mother or sister had an operation due to inguinal hernia or varicose veins?
    - o Yes
    - o No
    - o I do not know

General health

7. In general, how would you say your health is?
    - o Excellent
    - o Very good
    - o Good
    - o Fair
    - o Poor

Breastfeeding (at 8–10 weeks and 1 year postpartum only)

8. Do you breastfeed your child?
    - Yes, I breastfeed my child completely
    - Yes, I breastfeed my child partially
    - No, I do not breastfeed my child

Menstruation (at 8–10 weeks and 1 year postpartum only)

9. Have your monthly periods come back after delivery?
    - Yes, I have regular periods
    - Yes, I have irregular periods
    - No, my periods have not come back

Contraception (at 8–10 weeks and 1 year postpartum only)

10. Do you and your partner use any contraceptives?
    - Yes, I use a hormonal method (e.g. contraceptive pills, mini pills)
    - Yes, I have a coil
    - Yes, we use a barrier method (e.g. condoms)
    - No, we do not use any contraceptives

Smoking (questions 12–15 only when registering at the maternity health care)

11. Do you smoke?
    - Yes If yes, proceed to question 15
    - No
12. Have you smoked previously?
    - Yes If yes, proceed to question 13
    - No
13. When did you smoke your last cigarette? Year…………………
14. For how many years had you been smoking then? Number of years……….
15. When did you start to smoke? Year…………..
16. How many cigarettes do you smoke each day at present? ……………….

Urinary symptoms

17. Do you occasionally experience urinary leakage?
    - No If no, proceed to question 23
    - Yes

If yes, how often does urinary leakage bother you? (this question only when registering at the maternity health care)

- Less than once a month
- Once a month or more
- Once a week or more
- Every day and/or night

If yes, how much does it bother you? (this question only in late pregnancy, at 8–10 weeks postpartum, and at 1 year postpartum)

- Not at all
- Slightly
- Moderately
- Greatly

18. What amount of urine do you leak on each occasion?
    - A few drops or just a little
    - Small amounts
    - Large amounts
19. Do you occasionally experience a sudden need to urinate and then have difficulty reaching the toilet in time?
    - Yes, often
    - Sometimes
    - Infrequently
    - No, never
20. Do you leak urine when coughing, sneezing, lifting, or during physical activities?
    - Yes, often
    - Sometimes
    - Infrequently
    - No, never
21. Did you experience urinary leakage before pregnancy?
    - Yes, I experienced urinary leakage before pregnancy
    - Urinary leakage started during pregnancy
    - Urinary leakage started after delivery

Bowel habits

22. How often do you pass a bowel motion?
    - Daily
    - Every 1–2 days
    - Every 1–3 days
    - 1–2 times a week
    - Less than once a week

- I do not know

23. Do you ever experience overly-hard stools?
    - Never
    - Seldom
    - Sometimes
    - Often, practically every time
24. Do you ever experience incomplete evacuation of the bowels?
    - No
    - Yes

    If yes, how much does it bother you?

    - Not at all
    - Slightly
    - Moderately
    - Greatly
25. Do you have to digitally manipulate your vagina, perineum, or anus to achieve complete evacuation of the bowels?
    - No
    - Yes

    If yes, how much does it bother you?

    - Not at all
    - Slightly
    - Moderately
    - Greatly
26. Do you occasionally involuntarily leak gas?
    - No
    - Yes

    If yes, how much does it bother you?

    - Not at all
    - Slightly
    - Moderately
    - Greatly
27. Do you occasionally experience solid stool leakage?
    - No
    - Yes

    If yes, how much does it bother you?

    - Not at all
    - Slightly
    - Moderately
    - Greatly

28. Do you occasionally experience liquid stool leakage?
    - No
    - Yes

    If yes, how much does it bother you?

    - Not at all
    - Slightly
    - Moderately
    - Greatly

Symptoms of prolapse

29. Do you have a sensation of tissue protruding from your vagina (vaginal bulge)?
    - Yes, often
    - Sometimes
    - Infrequently
    - No, never
30. Do you suffer from scraping in your vagina/vulva?
    - Yes, often
    - Sometimes
    - Infrequently
    - No, never
31. Do you have to lift your anterior vaginal wall to start or complete voiding?
    - Yes, often
    - Sometimes
    - Infrequently
    - No, never

If you answered “Yes” or “Sometimes” to any of the questions above, please answer the following question as well:

32. Do your symptoms become worse during physical strain, for example lifting heavy objects?
    - Unchanged
    - Better
    - Worse

Quality of life

Do any symptoms from the pelvic floor bother you? (urinary leakage, gas/stool leakage, prolapse)

- Yes
- No, If no, please proceed to question 40

If yes, to what extent do those symptom affect your:

33. Ability to do household chores (cooking, housecleaning, laundry)?
    - Not at all
    - Slightly
    - Moderately
    - Greatly

34. Physical recreation such as walking, swimming, or other exercise?
    - Not at all
    - Slightly
    - Moderately
    - Greatly

35. Entertainment activities (movies, concerts, etc.)?
    - Not at all
    - Slightly
    - Moderately
    - Greatly

36. Ability to travel by car or bus more than 30 minutes from home?
    - Not at all
    - Slightly
    - Moderately
    - Greatly

37. Participation in social activities outside your home?
    - Not at all
    - Slightly
    - Moderately
    - Greatly

38. Emotional health (nervousness, depression, etc)?
    - Not at all
    - Slightly
    - Moderately
    - Greatly

39. Feeling frustrated?
    - Not at all
    - Slightly
    - Moderately
    - Greatly

Sex life

Following are a list of questions about you and your partner's sex life. All information is strictly confidential. Your confidential answers will be used only to help doctors understand what is important to patients about sex lives. Please check the box that best answers the questions for you.
While answering the questions, consider your sexuality over the past six months.

Are you sexually active?
- Yes
- No, If no, please proceed to question 52

40. How frequently do you feel sexual desire? This feeling may include wanting to have sex, planning to have sex, feeling frustrated due to lack of sex, etc.
    - Always
    - Usually
    - Sometimes
    - Seldom
    - Never

41. Do you climax (have an orgasm) when having sexual intercourse with your partner?
    - Always
    - Usually
    - Sometimes
    - Seldom
    - Never

42. Do you feel sexually excited (turned on) when having sexual activity with your partner?
    - Always
    - Usually
    - Sometimes
    - Seldom
    - Never

43. How satisfied are you with the variety of sexual activities in your current sex life?
    - Always
    - Usually
    - Sometimes
    - Seldom
    - Never

44. Do you feel pain during sexual intercourse?
    - Always

- Usually
- Sometimes
- Seldom
- Never

45. Are you incontinent of urine (leak urine) with sexual activity?
    - Always
    - Usually
    - Sometimes
    - Seldom
    - Never

46. Does fear of incontinence (either stool or urine) restrict your sexual activity?
    - Always
    - Usually
    - Sometimes
    - Seldom
    - Never

47. Do you avoid sexual intercourse because of bulging in the vagina (either the bladder, rectum or vagina falling out)?
    - Always
    - Usually
    - Sometimes
    - Seldom
    - Never

48. When you have sex with your partner, do you have negative emotional reactions such as fear, disgust, shame or guilt?
    - Always
    - Usually
    - Sometimes
    - Seldom
    - Never

49. Does your partner have a problem with erections that affects your sexual activity?
    - Always
    - Usually
    - Sometimes
    - Seldom
    - Never

50. Does your partner have a problem with premature ejaculation that affects your sexual activity?
    - Always

- Usually
- Sometimes
- Seldom
- Never

51. Compared to orgasms you have had in the past, how intense are the orgasms you have had in the past six months?
    - Much less intense
    - Less intense
    - Same intensity
    - More intense
    - Much more intense

Miscellaneous questions

52. Do you have any other pelvic floor symptoms that are not mentioned above? (Several options may be chosen)
    - Impaired ability to contract the pelvic floor
    - Gas in vagina during sexual intercourse or physical training
    - Sensation of impaired genital feeling
    - Sensation of wide vagina
    - Feeling of deficient support of the rear vaginal wall toward the rectum
    - Other……………………………………………….

Thank you for participating!
