# Supplementary material for: Risk factors for perineal and vaginal tears in primiparous women – the prospective POPRACT-cohort study
Source: BMC Pregnancy Childbirth. 2020 Dec 2;20:749. doi: 10.1186/s12884-020-03447-0 (PMC7709229; doi:10.1186/s12884-020-03447-0)
Supplement: Supplementary file 2 — Additional file 2. [file 12884_2020_3447_MOESM2_ESM.pdf]

Table. Unadjusted and adjusted odds ratios for risk factors for perineal tear including women being subject to episiotomy.

n=489	2nd degree		OASI	
	OR (95% CI)	aOR (95% CI)	OR (95% CI)	aOR (95% CI)
**Age**				
≤25 years	Reference	Reference	Reference	Reference
>25 years	2.01 (1.21, 3.35)*	1.89 (1.07, 3.35)*	1.56 (0.57, 4.25)	1.67 (0.51, 5.49)*
**BMI**				
≤25 kg/m^2	Reference	Reference	Reference	Reference
25.1–30 kg/m^2	0.91 (0.58, 1.42)	1.00 (0.62, 1.62)	1.09 (0.47, 2.51)	0.97 (0.38, 2.45)
>30kg/m^2	1.22 (0.68, 2.19)	1.18 (0.62, 2.24)	0.55 (0.12, 2.50)	0.28 (0.05, 1.48)
**Education**				
9to <12years	0.24 (0.03, 2.20)	0.36 (0.04, 3.54)	3.22 (0.56, 18.58)	4.53 (0.61, 33.95)
12 years	0.82 (0.55, 1.21)	0.92 (0.6, 1.42)	0.63 (0.27, 1.47)	0.61 (0.23, 1.6)
University	Reference	Reference	Reference	Reference
**Heredity**[a]				
No	Reference	NE	Reference	NE
Yes	1.33 (0.76, 2.32)	NE	1.52 (0.53, 4.35)	NE
**SUI in late pregnancy**				
No	Reference	NE	Reference	NE
Yes	0.66 (0.41, 1.05)	NE	1.65 (0.74, 3.66)	NE
**GA at birth**				
Preterm/term	Reference	Reference	Reference	Reference
Postterm	2.11 (1.09, 4.06)*	1.8 (0.81, 4.04)	2.01 (0.63, 6.48)	1.22 (0.28, 5.26)
**Delivery start**				
Spontaneous	Reference	Reference	Reference	Reference
Induction	1.38 (0.86, 2.21)	0.96 (0.54, 1.74)	1.6 (0.67, 3.81)	1.33 (0.45, 3.91)
**Epidural analgesia**				
No	Reference	Reference	Reference	Reference
Yes	1.76 (1.21, 2.55)*	0.8 (0.53, 1.2)	2.06 (0.98, 4.34)*	0.84 (0.33, 2.11)
**Oxytocin stimulation**				
No	Reference	Reference	Reference	Reference
Yes	1.76 (1.21, 2.55)*	1.22 (0.78, 1.92)	2.06 (0.98, 4.34)*	0.84 (0.33, 2.11)
**Duration of active 2nd stage**				
≤15 min	0.74 (0.45, 1.23)	0.77 (0.45, 1.31)	1.2 (0.49, 2.92)	1.52 (0.57, 4.03)
16–60 min	Reference	Reference	Reference	Reference
>60 min	1.23 (0.80, 1.92)	1.08 (0.67, 1.72)	0.8 (0.30, 2.11)	0.55 (0.19, 1.57)
**Maternal position at birth**				
Flexible sacrum positions	Reference	Reference	Reference	Reference
Reduced sacrum flexibility	0.94 (0.59, 1.49)	0.60 (0.36, 1.00)	1.07 (0.42, 2,75)	0.67 (0.23, 1.98)
**Mode of delivery**				
Spontaneous	Reference	Reference	Reference	Reference
Vacuum				

extraction	3.37 (1.92, 5.93)*	3.40 (1.83, 6.31)	4.92 (2.04, 11.84)*	4.97 (1.79, 13.83)*
**Fetal scalp electrode**				
No	Reference	Reference	Reference	Reference
Yes	1.44 (0.99, 2.08)	1.26 (0.82, 1.92)	2.76 (1.23, 6.19)*	2.41 (0.98, 5.94)
**Fetal presentation**				
Occiput anterior	Reference	Reference	Reference	Reference
Occiput posterior	1.37 (0.50, 3.74)	1.45 (0.5, 4.19)	3.24 (0.80, 13.22)	4.05 (0.83, 19.7)
**Hand or arm presenting fetal part**				
No	Reference	NE	Reference	NE
Yes	0.99 (0.55, 1.76)	NE	0.77 (0.22, 2.68)	NE
**Fetal weight**				
≤4000 g	Reference	Reference	Reference	Reference
>4000 g	2.56 (1.44, 4.57)*	2.31 (1.25, 4.29)*	5.66 (2.39, 13.40)*	6.00 (2.32, 15.50)*
**Fetal head circumference**				
≤35 cm	Reference	NE	Reference	NE
>35 cm	1.91 (1.31, 2.77)*	NE	3.58 (1.55, 8.27)*	NE

Unadjusted and adjusted odds ratios for risk factors for perineal tear using multinomial logistic regression. The group of women with second-degree perineal tear and OASI were compared with women with no or first-degree perineal tear. Women having an episiotomy were included in this analysis. [a]Heredity of pelvic floor dysfunction and/or connective tissue deficiency; *Significant at level $p<0.05$. aOR, adjusted odds ratio; BMI, body mass index; CI, confidence interval; GA, gestational age; NE, not estimated; OASI, obstetric anal sphincter injury; OR, odds ratio; SUI, stress urinary incontinence.
